# Tumor mutation burden estimated by a 69-gene-panel is associated with overall survival in patients with diffuse large B-cell lymphoma

**DOI:** 10.1186/s40164-021-00215-4

**Published:** 2021-03-15

**Authors:** Cunte Chen, Sichu Liu, Xinmiao Jiang, Ling Huang, Feili Chen, Xiaojun Wei, Hanguo Guo, Yang Shao, Yangqiu Li, Wenyu Li

**Affiliations:** 1 Institute of Hematology, School of Medicine, Key Laboratory for Regenerative Medicine of Ministry of Education, Jinan University, Guangzhou, China; 2 Department of Lymphoma, Guangdong Provincial People’s Hospital, Guangdong Academy of Medical Sciences, School of Medicine, South China University of Technology, Guangzhou, China; 3 Nanjing Geneseq Technology Inc, Nanjing, Jiangsu China; 4 School of Public Health, Nanjing Medical University, Nanjing, Jiangsu China

**Keywords:** TMB, Gene panel, Prognosis, Biomarker, Diffuse large B-cell lymphoma

## Abstract

**Background:**

Tumor mutation burden (TMB) as estimated by cancer gene panels (CGPs) has been confirmed to be associated with prognosis and is effective in predicting clinical benefit from immune checkpoint blockade (ICB) in solid tumors. However, whether the TMB calculated by CGPs is associated with overall survival (OS) for patients with diffuse large B-cell lymphoma (DLBCL) is worth exploring.

**Methods:**

The prognostic value of panel-TMB, calculated by a panel of 69 genes (GP69), for 87 DLBCL patients in our clinical center (GDPH dataset) was explored. The results were further validated using 37 DLBCL patients from the Cancer Genome Atlas (TCGA) database (TCGA dataset).

**Results:**

Spearman correlation analysis suggested that panel-TMB is positively correlated with the TMB calculated by whole-exome sequencing (wTMB) in the TCGA dataset (R = 0.76, P < 0.0001). Both GDPH and TCGA results demonstrated that higher panel-TMB is significantly associated with a poor OS for DLBCL patients (P < 0.05) where a panel of 13 genes was associated with poor OS, and another panel of 26 genes was correlated with a favorable OS for DLBCL patients. Further subgroup analysis indicated that higher panel-TMB had shorter OS in DLBCL patients with younger than 60 years, elevated LDH, greater than one extranodal involvement, stage III/IV, an IPI score of 3–5, or HBsAg, anti-HBc, or HBV-DNA negativity (P < 0.05). Interestingly, the nomogram model constructed by panel-TMB, stage, and IPI could individually and visually predict the 1-, 2- and 3-year OS rates of DLBCL patients.

**Conclusions:**

We established GP69 for the evaluation of OS for Chinese DLBCL patients. panel-TMB might be a potential predictor for prognostic stratification of DLBCL patients.

**Supplementary Information:**

The online version contains supplementary material available at 10.1186/s40164-021-00215-4.

## Introduction

Diffuse large B-cell lymphoma (DLBCL) is the most common type of aggressive non-Hodgkin lymphoma, which can occur de novo or is caused by the transformation of indolent lymphoma [1–3]. A large number of patients can be clinically relieved or even cured using standard chemo-immunotherapy; however, approximately one-third of patients still have a poor prognosis due to drug resistance or relapse, which is partially related to the heterogeneity of DLBCL [4–7]. This heterogeneity is manifested at the clinical level and in the morphology, genetics, and immunophenotype. However, the current prognostic scoring system stratifies DLBCL patients based on an international prognostic index (IPI) of clinical level, including age, stage, performance status (PS), serum lactate dehydrogenase (LDH) level, and the amount of extranodal involvement [8–10]. In fact, through next-generation sequencing (NGS) analysis of DLBCL, mutations in a number of genes that play a crucial role in tumor progression, maintenance, and response to treatment have been discovered [1]. Therefore, there is an urgent need for prognostic stratification of DLBCL patients based on mutations to guide clinical treatment.

Tumor mutation burden (TMB) is the number of somatic mutations per megabase (Mb) of the genome in a tumor, representing the instability in its genome. Tumors with high TMB are more likely to induce neoantigens' production, making them a target of activated immune cells [11, 12]. Recent studies have shown that high TMB measured by whole-exome sequencing (WES) is closely related to higher response rates to ICB in cancers, thereby predicting favorable clinical outcomes [13, 14]. However, because of the cost of whole-genome sequencing (WES), the timeliness and informatics challenges of WES in the clinical setting, it is difficult to popularize in clinical applications [15, 16]. Instead, it is now clinically more common to use a smaller cancer gene panel (CGP) for precise therapy, immunotherapy, and patients' prognostic stratification [15, 17, 18].

Hence, in this study, the previously reported lymphoma-related genes [19] overlapped with the WES data in the Cancer Genome Atlas (TCGA) database, plus hot spot mutation genes, 69 genes were obtained for developing a panel for TMB estimation (panel-TMB), which could be used for overall survival (OS) analysis. We further validated our findings using data from the TCGA database.

## Materials and methods

### Patient samples

**Fig. 1:**
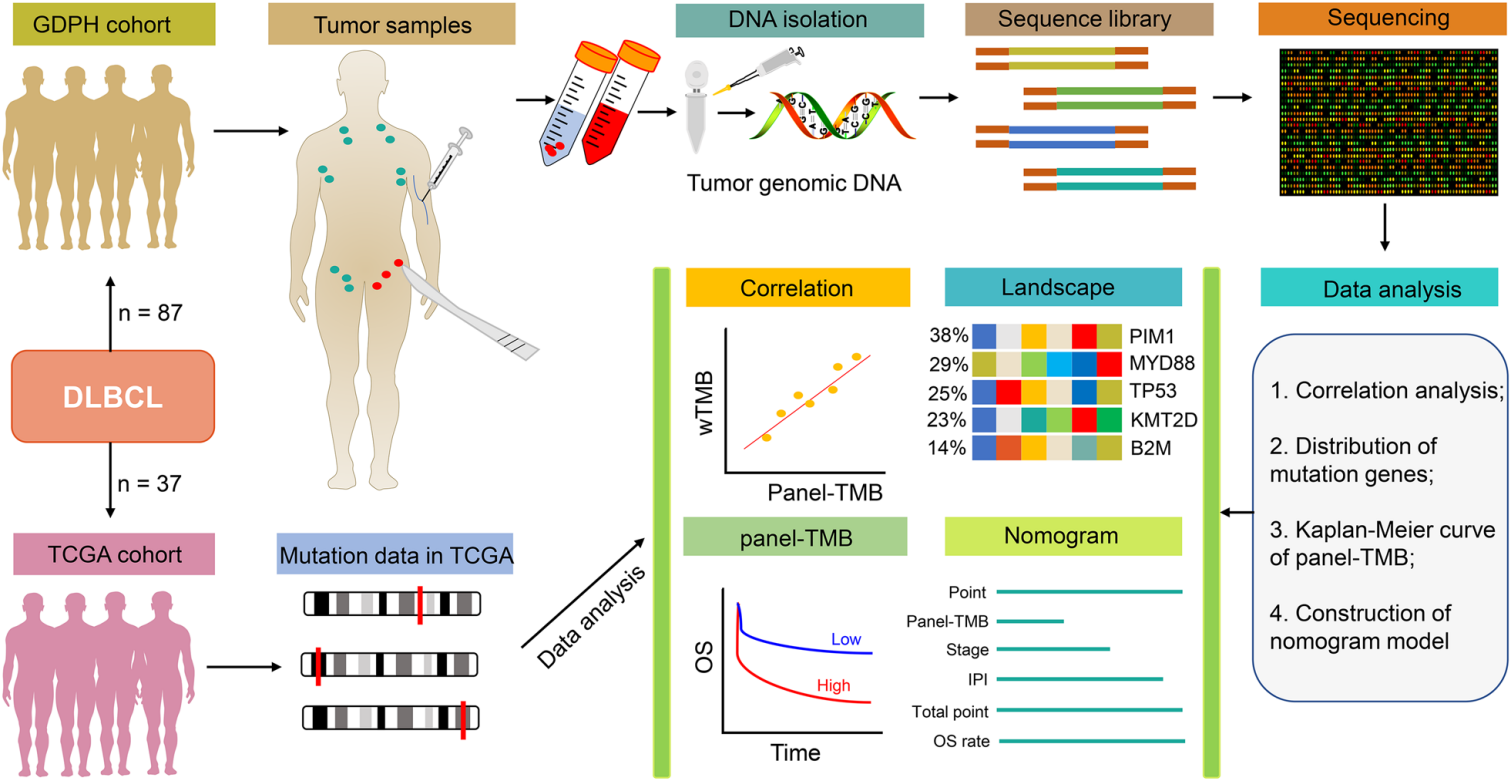
Workflow of study. A total of 87 DLBCL patients from our clinical center were designated as a GDPH cohort, and their whole blood and tumor biopsies were obtained to isolate genomic deoxyribonucleic acid (DNA). Construction of DNA sequence library for exon sequencing and data mining. Furthermore, the UCSC-XENA platform (https://xenabrowser.net/datapages/) was used to download the whole-exome sequencing data of 37 DLBCL patients in the Cancer Genome Atlas (TCGA) database for data analysis. The mutation frequency and type of a panel of 69 genes (GP69) and the relationship between panel-tumor mutation burden (panel-TMB) calculated by GP69 and TMB estimated by whole-exome sequencing (wTMB) and prognosis were investigated. GDPH, Guangdong Provincial People's Hospital

A total of 87 whole blood and tumor biopsies were collected from patients newly diagnosed with DLBCL at Guangdong Provincial People’s Hospital (GDPH) between January 21, 2014, and July 15, 2019, for use with targeted sequencing using the GP69 panel [20]. Clinical characteristics including gender, age, immunophenotype, LDH level, extranodal involvement, eastern cooperative oncology group performance status (ECOG PS), Ann Arbor stage, IPI score, double-hit and double-express or lymphomas (DHL/DEL), hepatitis B surface antigen (HBsAg), antibody to hepatitis B core antigen (anti-HBc), and hepatitis B virus DNA (HBV-DNA) status, and treatment options data (Table 1). The last follow-up was completed on May 20, 2020, and the median follow-up time for the DLBCL patients was 435 days (range 5–1722 days). OS was defined as the time from diagnosis to death of any cause or last follow-up. The workflow of data mining in the GDPH cohort was shown in Fig. 1. This study was performed according to the Declaration of Helsinki principles and approved by the Ethics Committee of Guangdong Provincial People’s Hospital. All participants provided written informed consent.

**Table 1:** Clinical characteristics of DLBCL patients in the GDPH and TCGA datasets (n = 124)

Variables	GDPH dataset, n (%)^a^	TCGA dataset, n (%)^a^	P value
Number	87	37	
Gender			0.528
Female	44 (50.6)	21 (56.8)	
Male	43 (49.4)	16 (43.2)	
Age, years			0.638
Younger than 60	51 (58.6)	20 (54.1)	
Older than 60	36 (41.4)	17 (45.9)	
Immunophenotype			NA
GCB	24 (27.6)	–	
Non-GCB	46 (52.9)	–	
Unclassified	2 (2.3)	–	
Unknown	15 (17.2)	–	
Serum LDH level			< 0.001
Normal	31 (35.6)	12 (32.4)	
Elevated	56 (64.4)	12 (32.4)	
Unknown	0 (0.0)	13 (35.1)	
Extranodal involvement			< 0.001
0–1	47 (54.0)	17 (45.9)	
More than 1	40 (46.0)	10 (27.0)	
Unknown	0 (0.0)	10 (27.0)	
ECOG PS			NA
0–1	66 (75.9)	–	
2–4	21 (24.1)	–	
Ann Arbor stage			< 0.001
I/II	33 (37.9)	21 (56.8)	
III/IV	54 (62.1)	11 (29.7)	
Unknown	0 (0.0)	5 (13.5)	
IPI score			NA
0–2	46 (52.9)	–	
3–5	41 (47.1)	–	
DHL/DEL		–	NA
Yes	30 (34.5)	–	
No	57 (65.5)	–	
HBsAg		–	NA
Positive	17 (19.5)	–	
Negative	70 (80.5)	–	
Anti-HBc		–	NA
Positive	18 (20.7)	–	
Negative	69 (79.3)	–	
HBV-DNA		–	NA
Positive	10 (11.5)	–	
Negative	77 (88.5)	–	
Treatment			NA
R-CHOP	74 (85.1)	–	
BFM-90	1 (1.1)	–	
Rituximab + Lenalidomide	7 (8.0)	–	
Radiation	0 (0)	6 (16.2)	
Unknown	5 (5.7)	1 (2.7)	

^a^Due to rounding, not all percentages total 100%

*anti-HBc* antibody to hepatitis B core antigen, *BFM-90* Berlin–Frankfurt–Munster-90 regimen, *DHL/DEL* double-hit and double-expressor lymphomas, *ECOG PS* Eastern Cooperative Oncology Group performance status, *GDPH* Guangdong Provincial People’s Hospital, *GCB* germinal center B cells, *HBsAg* hepatitis B surface antigen, *HBV-DNA* hepatitis B virus DNA, *IPI* international prognostic index, *LDH* lactate dehydrogenase, *NA* not available, *R-CHOP* rituximab, cyclophosphamide, adriamycin, vincristine, and prednisone

### Library construction

Tumor genomic DNA was extracted from whole blood and tumor biopsies, and fragmented DNA was generated with a Bioruptor (Diagenode, Bioruptor UCD-200) following the manufacturer's protocol. Libraries were constructed using the KAPA Hyper DNA Library Prep Kit (KAPA Biosystem, KK8504). Finally, dual-indexed sequencing libraries were PCR amplified with KAPA HiFi Hot start-ready Mix (KAPA, KK2602) for 4–6 cycles, and they were then cleaned up with purification Beads (Corning, AxyPrep Fragment Select-I kit, 14223162). The library concentration and quality were determined using the Qubit 3.0 system (Invitrogen) and Bioanalyzer 2100 (Agilent, Agilent HS DNA Reagent, 5067-4627).

### Hybrid selection and ultra-deep next-generation sequencing

A 5′-biotinylated probe solution was used as capture probes. The probes for targeted sequencing covered exons and selected introns in 69 DLBCL-related genes (Additional file 1: Table S1) in a cohort of 87 patients. A total of 1 μg of each fragmented sequencing library was mixed with 5 μg salmon sperm DNA, 5 μg human Cot-1 DNA, and 1unit adaptor-specific blocker DNA in hybridization buffer and then heated for 10 min at 95 °C and held for 5 min at 65 °C in a thermocycler. The capture probes were added to the mixture in 5 min, and solution hybridization was performed for 16–18 h at 65 °C. After hybridization was complete, the captured targets were selected by pulling down the biotinylated probe/target hybrids using streptavidin-coated magnetic beads, and the off-target library was removed using wash buffer. PCR master mix was directly added to amplify (6–8 cycles) the captured library from the washed beads. Afterward, the samples were purified by AMPure XP beads, quantified by qPCR (Kapa), and sized with a bioanalyzer 2100 (Agilent, Agilent HS DNA Reagent, 5067-4627). Libraries were normalized to 2.5 nM and then pooled. Finally, the library was sequenced as paired 150 bp reads with an Illumina HiSeq 4000 according to the manufacturer's instructions.

### Sequence alignment and processing

Base-calling was performed using bcl2fastq v2.16.0.10 (Illumina, Inc.) to generate sequence reads in FASTQ format (Illumina 1.8 + encoding). Quality control (QC) was performed with Trimmomatic. High-quality reads were mapped to the human genome (hg19, GRCh37 Genome Reference Consortium Human Reference 37) using the BWA aligner 0.7.12 with the BWA-MEM algorithm using default parameters to create SAM files. Picard 1.119 was used to convert SAM files to compressed BAM files, which were then sorted according to chromosome coordinates. The genome analysis tool kit (GATK, version 3.4-0) was used to locally realign the BAM files at loci with indel mismatches and recalibrate the base quality scores of the reads in the BAM files.

### SNV/indel/CNV detection

SNVs and short Indels were identified by VarScan2 2.3.9 with the minimum variant allele frequency threshold set at 0.01 and the p-value threshold for calling variants set at 0.05 to generate variant call format (VCF) files. All SNVs/indels were annotated with ANNOVAR, and each SNV/indel was manually checked in the Integrative Genomics Viewer (IGV). Copy number variations (CNVs) were detected using in-house-developed software.

### TCGA dataset

The non-synonymous mutation data of 37 de novo DLBCL patients in the TCGA database (https://cancergenome.nih.gov/) were downloaded using the UCSC XENA platform (https://xenabrowser.net/datapages/) [21–23]. The Multiple Cancers (MC3) project was used for the mutation calling of tumor exomes in this study. Somatic mutations mainly included single nucleotide variants (SNVs) and insertions/deletions (INDELs). The clinical information of the 37 DLBCL patients from the TCGA database was listed in Table 1. The TCGA database is publicly available; thus, approval from the local ethics committee was not required.

### Statistical analysis

All statistical analyses were conducted with SPSS (version 22.0, IBM, Armonk, NY, USA) and R (version 3.6.1, https://www.r-project.org/) as appropriate. The optimal cut-off value for panel-TMB was determined using maximally selected rank statistics in the "maxstat" R package [24, 25], which was reflected in the "survminer" package. The log-rank test was used to compare differences between Kaplan–Meier curves. The coefficients of the univariate COX regression model were acquired by SPSS 22.0 software. The Spearman method was applied to obtain correlation coefficients between two groups of quantitative variables. Differences in qualitative variables were compared by the chi-square test. A nomogram model was constructed based on the previous study [26]. The "survRM2" package was used to determine restricted mean survival time (RMST). A two-tailed P value < 0.05 was considered statistically significant.

## Results

### The relationship between panel-TMB and tumor mutation burden estimated by whole-exome sequencing (wTMB)

**Fig. 2:**
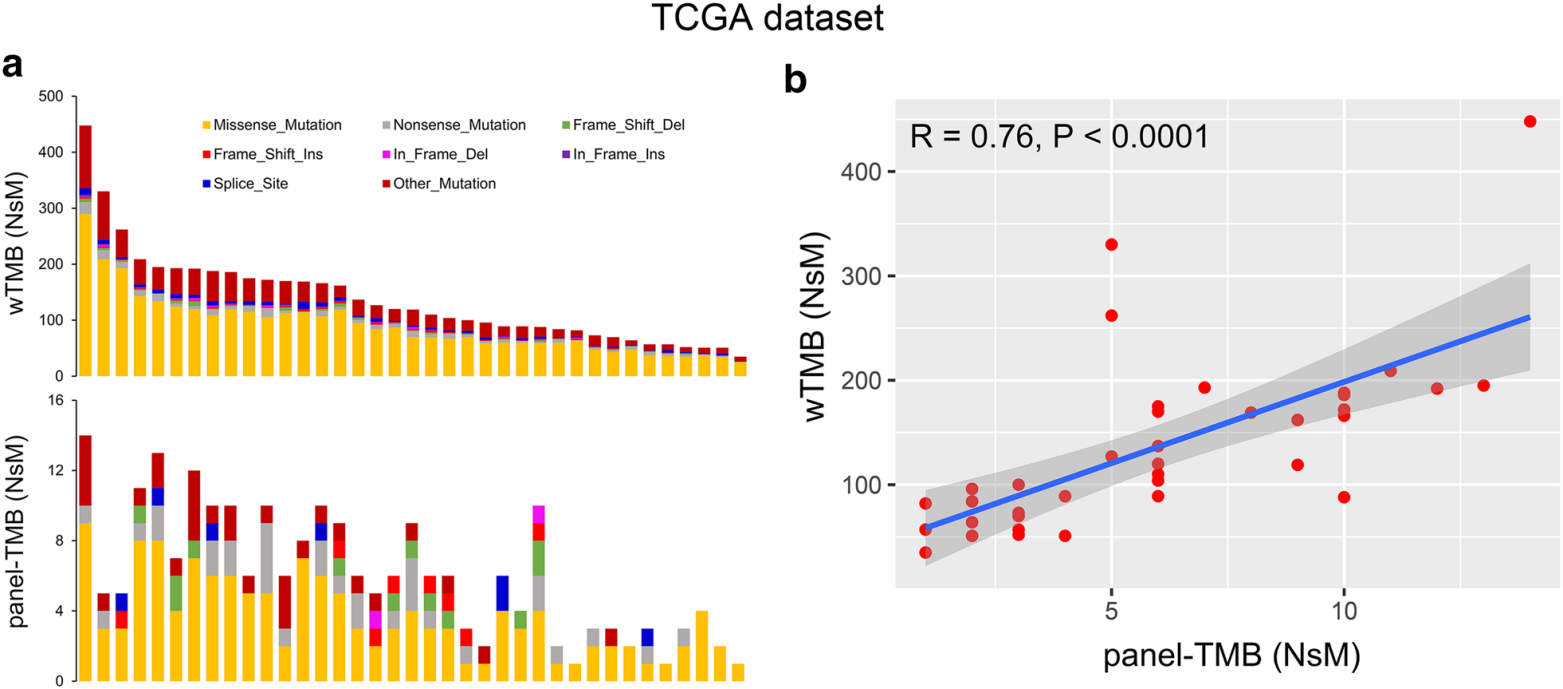
The relationship between panel-TMB and the tumor mutation burden estimated by whole-exome sequencing (wTMB) in the TCGA dataset. **a** The distribution of NsMs was obtained by whole-exome sequencing (upper panel) and a 69-gene panel (lower panel) for 37 DLBCL patients. **b** panel-TMB and wTMB demonstrated a significant positive correlation in 37 DLBCL patients. R, Spearman correlation coefficient

To evaluate whether panel-TMB can reflect wTMB, 37 DLBCL patients in the TCGA dataset were used to analyze the correlation between the two. As shown in Fig. 2a, non-synonymous mutations (NsMs) derived from whole-exome sequencing and GP69 are relatively consistent in DLBCL patients. Further, Spearman correlation analysis found that panel-TMB and wTMB have a significant positive correlation (R = 0.76, P < 0.0001, Fig. 2b). These results indicated that the GP69 developed by us could well represent the wTMB in DLBCL patients.

### The landscape of DLBCL-GP69

**Fig. 3:**
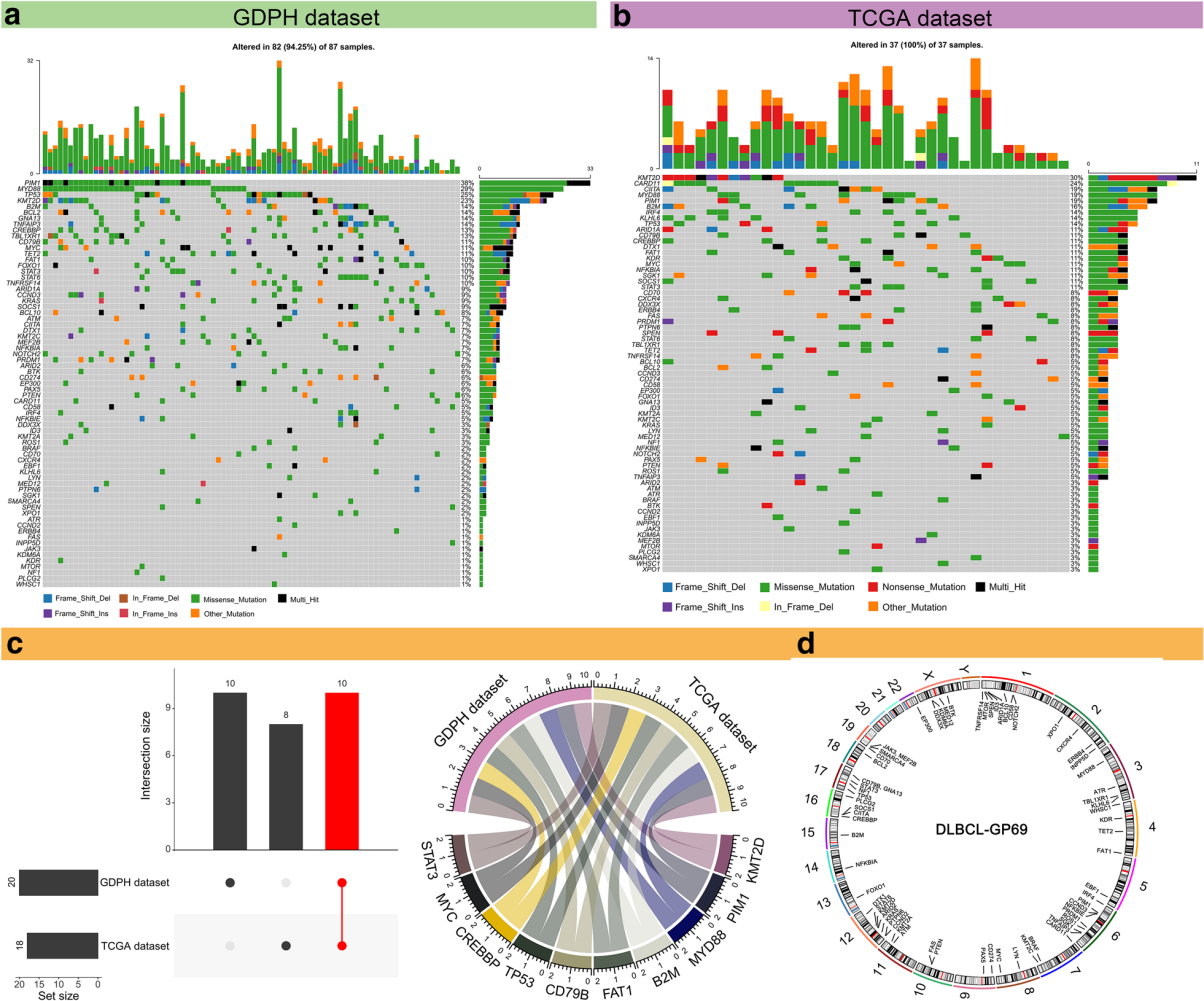
The mutational landscape of 69 genes in patients with diffuse large B cell lymphoma (DLBCL). **a**, **b** Mutation landscape for the 69 genes in the GDPH (**a**) and TCGA (**b**) datasets. The histogram above each plot showing the number of non-synonymous mutations (NsMs) in each patient, and the histogram on the right displays the number of patients with a mutation in each gene. **c** The overlap of genes whose mutation frequency is greater than 10% in the clinical and TCGA datasets is shown. The histogram shows the number of overlapping genes (left panel). The circos plot displays the overlapping genes (right panel). **d** The positions of the 69-panel genes on the chromosomes are shown. The outermost layer is the name of the chromosome, the second layer is the specific location of the gene, and the innermost layer is the name of the 69 genes. GP69, a panel composed of 69 genes

To visualize the distribution of mutations in the 69 genes for DLBCL patients, we depicted the landscape of DLBCL-GP69 in waterfall plots. Shown in Fig. 3a, b are the variant classifications of the 69 genes in the GDPH and TCGA datasets. We further analyzed genes with mutation frequencies greater than 10% and found the following ten genes in common in both the GDPH and TCGA datasets: KMT2D, PIM1, MYD88, B2M, FAT1, CD79B, TP53, CREBBP, MYC, and STAT3 (Fig. 3c). Besides, the GP69 genes were distributed on 21 chromosomes (Fig. 3d).

Next, the biological processes (BPs) of the GP69 genes were investigated. As shown in Additional file 2: Fig. S1, GP69 was involved in eight BPs, including apoptosis/cell proliferation, transcriptional regulation, cell cycle, chromatin modification, immune response, B cell receptor signaling pathway, JAK-STAT signaling pathway, and cell migration regulation, and the number of genes in each category was 26, 11, 7, 7, 6, 6, 4, and 2, respectively. These results suggested that the GP69 genes are primarily involved in important processes in tumor progression.

### Higher TMB estimated by a 69-gene panel (panel-TMB) is associated with poor OS

**Fig. 4:**
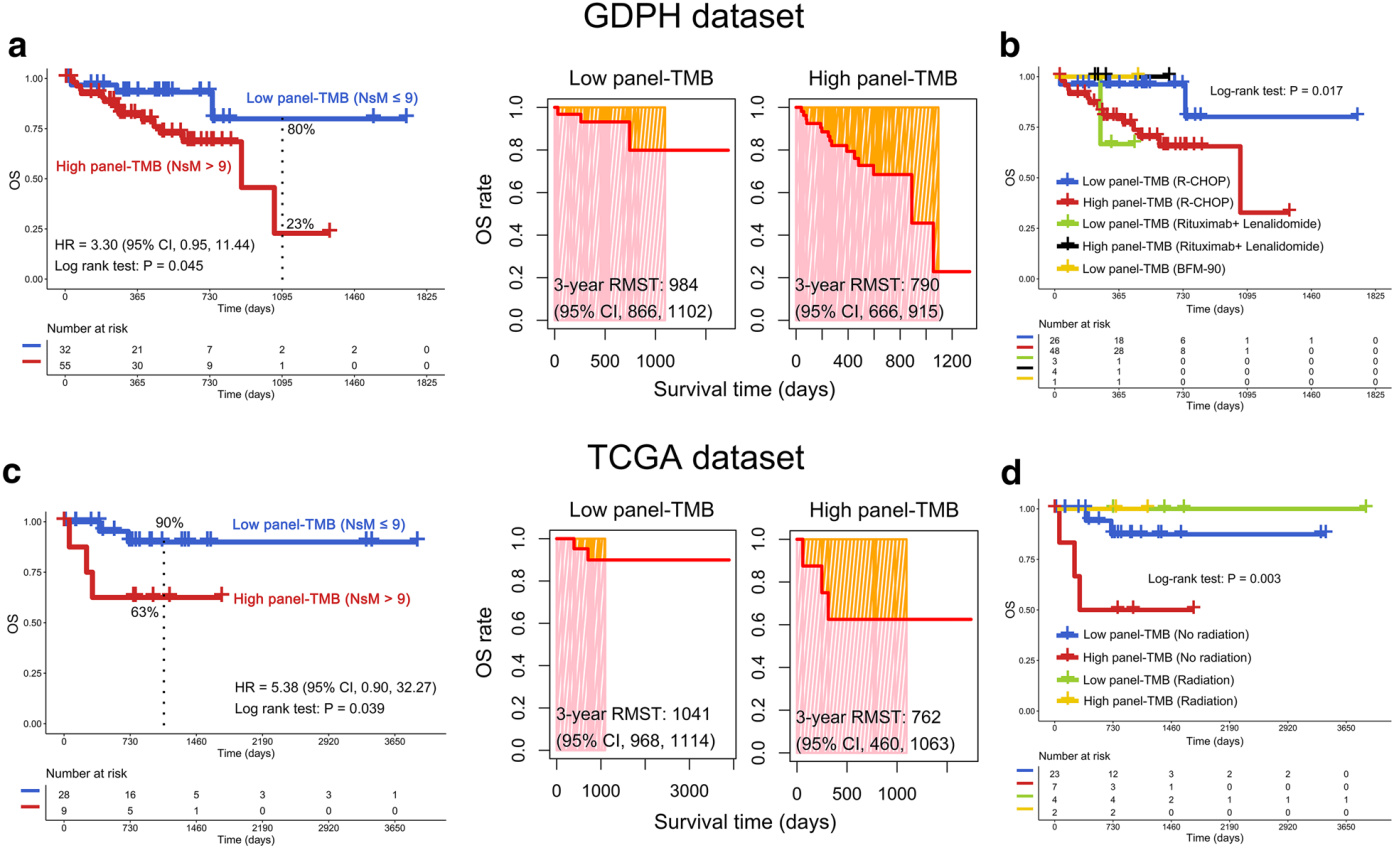
Overall survival (OS) analysis of tumor mutation burden as estimated by a 69-gene-panel (panel-TMB) in DLBCL patients. panel-TMB was associated with poor OS in the GDPH (**a**) and TCGA (**c**) datasets. Kaplan–Meier curves between low and high panel-TMB groups (left panel). The restricted mean survival time (RMST) was determined by "survRM2" package in R (version 3.6.1, https://www.r-project.org/) (right panel). The interaction of panel-TMB and treatment options in the GDPH (**b**) and TCGA (**d**) datasets

To better assess the impact of panel-TMB on the OS of patients with DLBCL, the optimal cut-off value for panel-TMB was determined. The cut-off values for panel-TMB in the GDPH and TCGA datasets were 4 and 9, respectively (Additional file 3: Fig. S2). A Kaplan–Meier curve demonstrated that DLBCL patients with higher panel-TMB predicted poor OS in the GDPH dataset (hazard ratio (HR) = 3.30, 95% confidence interval (CI), 0.95 to 11.44; 3-year OS 23% vs. 80%, P = 0.045) (Fig. 4a, left panel). This result was confirmed in the TCGA dataset (HR = 5.38, 95% CI, 0.90 to 32.27; 3-year OS: 63% vs. 90%, P = 0.039) (Fig. 4c, left panel). Moreover, patients with higher panel-TMB had a shorter RMST than those with low panel-TMB in the GDPH dataset (3-year RMST, 790 (95% CI, 666 to 915) vs. 984 (95% CI, 866 to 1102) days) (Fig. 4a, right panel). This result was again confirmed in the TCGA dataset (3-year RMST, 762 (95% CI, 460 to 1063) vs. 1041 (95% CI, 968 to 1114) days) (Fig. 4c, right panel). To determine whether treatment options impact results in terms of panel-TMB vs. OS, we performed an interaction analysis of panel-TMB and treatment options. The results showed that even when patients received different treatment options, high panel-TMB was still significantly associated with poor OS of DLBCL patients in both the GDPH and TCGA dataset (P = 0.017, P = 0.003, respectively) (Fig. 4b, d).

We further explored which genes had a greater contribution to OS for panel-TMB using a univariate COX regression model. A total of 13 genes were associated with poor OS (coefficient > 0): FAS, BCL2, CIITA, FOXO1, ROS1, SOCS1, CREBBP, PTEN, PAX5, TBL1XR1, MYD88, MYC, and PRDM1. Additionally, the following 26 genes were correlated with favorable OS (coefficient < 0): TNFAIP3, TNFRSF14, MEF2B, BCL10, BTK, CCND3, KMT2C, WHSC1, FAT1, PLCG2, JAK3, BRAF, DDX3X, STAT6, DTX1, KDR, ERBB4, ARID2, SMARCA4, KMT2A, SPEN, NFKBIE, XPO1, INPP5D, KDM6A, and MTOR. Among these genes, the mutation frequencies for MYD88, CREBBP, MYC, and FAT1 were greater than 10% (Additional file 4: Fig. S3A, B). Notably, we tried to streamline the gene number in the panel by using these 39 prognosis-related genes in both the GDPH and TCGA datasets. However, after reducing the number of genes in the panel, the calculated panel-TMB had no significant correlation with OS in both the GDPH and TCGA datasets (P > 0.05, Additional file 5: Fig. S4A, B). Therefore, panel-TMB estimated from 69 genes might be the minimal panel for OS analysis in this study.

### Panel-TMB subgroup analysis

**Fig. 5:**
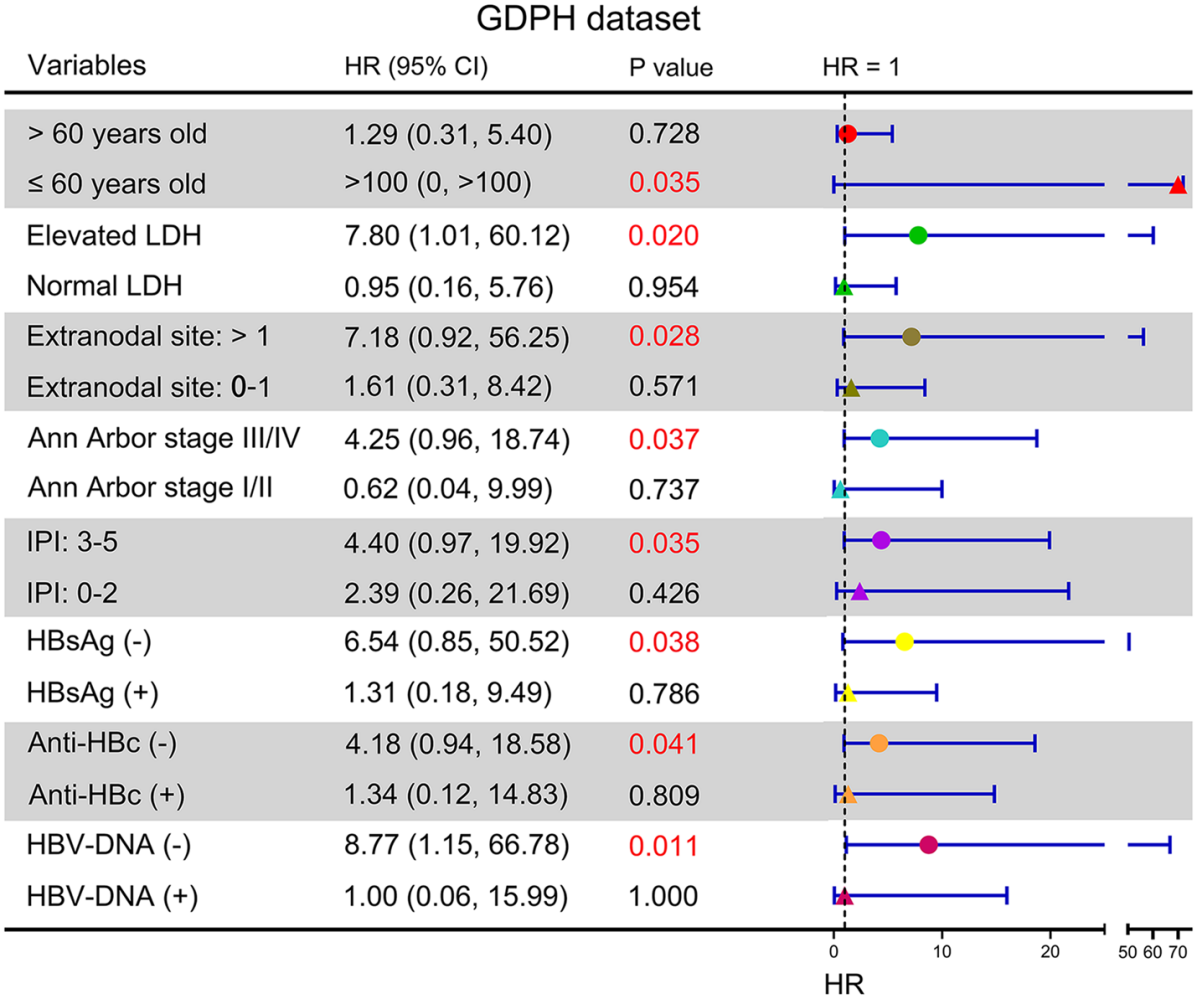
The effects of panel-TMB on OS in DLBCL patients of different ages, LDH levels, extranodal involvement, Ann Arbor stage, IPI, HBsAg, anti-HBc, and HBV-DNA in the GDPH dataset. LDH, lactate dehydrogenase; IPI, international prognostic index; HBsAg, hepatitis B surface antigen; anti-HBc, antibody to hepatitis B core antigen; HBV-DNA, hepatitis B virus DNA

To investigate the correlation between panel-TMB and clinical characteristics, we conducted a subgroup analysis of the GDPH dataset. As shown in Fig. 5, among DLBCL patients younger than 60 years, higher panel-TMB was correlated with poor OS (HR > 100, 95% CI, 0 to > 100, P = 0.035). When LDH levels were elevated, DLBCL patients with higher panel-TMB had shorter OS (HR = 7.80, 95% CI, 1.01 to 60.12, P = 0.020). In patients with greater than one extranodal involvement, higher panel-TMB might predict poor OS for DLBCL patients (HR = 7.18, 95% CI, 0.92 to 56.25, P = 0.028). When the patients were at stage III/IV, there was a difference in survival according to the level of panel-TMB (HR = 4.25, 95% CI, 0.96 to 18.74, P = 0.037). In patients with an IPI of 3–5, higher panel-TMB was significantly associated with poor OS for DLBCL patients (HR = 4.40, 95% CI, 0.97 to 19.92, P = 0.035). Moreover, the prognostic impact of panel-TMB for DLBCL patients with or without hepatitis B virus infection was analyzed. In patients with current (HBsAg or HBV-DNA positive) or past (anti-HBc) HBV infection, higher panel-TMB did not predict worse survival. When HBsAg and anti-HBc were negative, higher panel-TMB was associated with shorter OS (HR = 6.54, 95% CI, 0.85 to 50.52, P = 0.038, and HR = 4.18, 95% CI, 0.94 to 18.58, P = 0.041, respectively). Similarly, when the patients were negative for HBV-DNA, higher panel-TMB predicted shorter OS rates and survival time (HR = 8.77, 95% CI, 1.15 to 66.78, P = 0.011). However, the level of panel-TMB was not significantly correlated with OS for greater than 60 years of age, normal LDH, 0–1 extranodal involvement, stage I/II, IPI 0–2, sex, GCB subtype, non-GCB subtype, ECOG PS 0–1, ECOG PS 2–4, DHL/DEL, non-DHL/non-DEL, or HBsAg, anti-HBc, or HBV-DNA positivity (P > 0.05, Fig. 5 and Additional file 6: Fig. S5).

### Construction of nomogram model

**Fig. 6:**
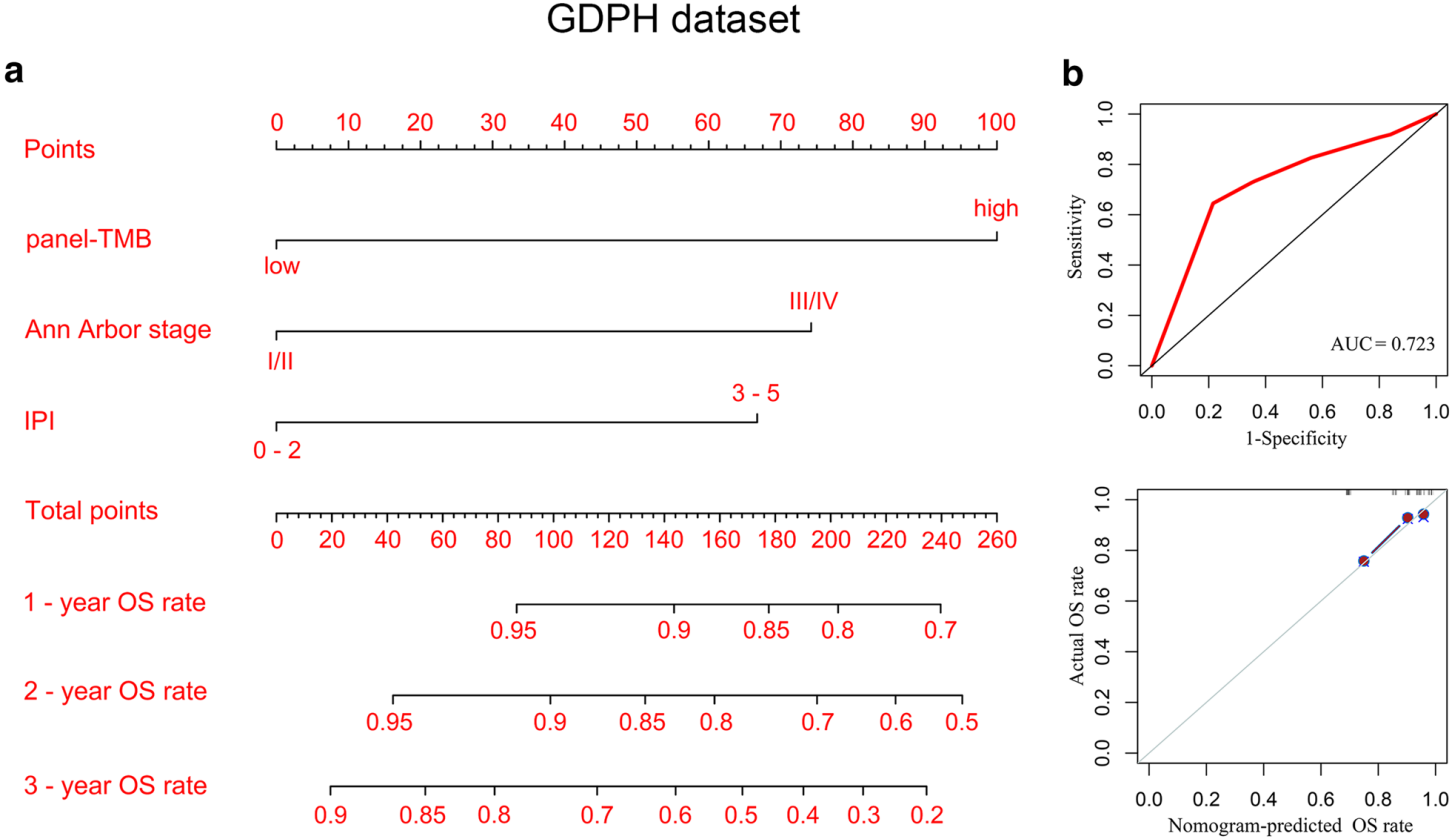
Construction of nomogram model in the GDPH dataset. **a** A combination of panel-TMB, Ann Arbor stage, and IPI visualized and personalized the OS rate of DLBCL patients. After the nomogram assigned a point for panel-TMB, stage, and IPI of each patient, the total points could be obtained to predict patients' OS rates. **b** The time-dependent receiver operating characteristic (ROC) (upper panel) and the calibration (bottom panel) curves were used to evaluate the performance of the nomogram model. AUC, the area under a curve

To visually and personally predict the OS rate of DLBCL patients, clinical information was used to construct the nomogram model. Kaplan–Meier curves showed that compared with stage I/II, stage III/IV was associated with poor OS of patients (P = 0.025, Additional file 7: Fig. S6). Similarly, a high IPI score predicted poor OS of DLBCL (P = 0.021, Additional file 7: Fig. S6). However, gender, age, LDH, extranodal involvement, ECOG PS, subtype, DHL/DEL, HBsAg, anti-HBc, or HBV-DNA was not significantly correlated with OS (P > 0.05, Additional file 7: Fig. S6). Thus, panel-TMB, stage, and IPI were used to construct a nomogram model for predicting 1-, 2- and 3-year OS rates of DLBCL patients (Fig. 6a), and the detailed points and OS rates were shown in Additional file 8: Table S2. Then, the time-dependent receiver operating characteristic (ROC) curve suggested that the nomogram model constructed by panel-TMB, stage, and IPI had a good performance (area under the curve (AUC) = 0.723) (Fig. 6b upper panel). Moreover, the calibration curve indicated that the OS rate predicted by the nomogram was in line with the actual OS rate (Fig. 6b bottom panel).

## Discussion

To develop an OS prediction system for Chinese DLBCL patients, in this study, we developed the GP69 panel, which includes genes distributed on 21 chromosomes that are mainly involved in tumor progression. Excitingly, we found a significant positive correlation between the panel-TMB measured by GP69 and the wTMB assessed by WES. These results suggest that the panel-TMB estimated by 69 genes involved in important BPs could replace wTMB in evaluating OS and represent the genomic instability in DLBCL patients. These findings are in accordance with studies in which CGPs were developed to replace WES to estimate TMB in cancer patients [27, 28].

To investigate the clinical significance of the panel-TMB estimated by GP69, we performed survival analysis of DLBCL patients. Firstly, we found that higher panel-TMB was significantly associated with a poor OS for DLBCL patients. Secondary, the nomogram model constructed by panel-TMB, stage, and IPI could individually and visually predict the 1-, 2- and 3-year OS rates of DLBCL. Interestingly, a previous study confirmed that higher TMB as estimated by a CGP is associated with a favorable prognosis and predicts the clinical benefits of ICB therapy [15, 29]. Moreover, in the absence of checkpoint inhibitor treatment, cancer patients with higher TMB tend to have adverse outcomes [30], which is consistent with our findings, thus, it may also indicate that higher panel-TMB might be an adverse prognostic factor for DLBCL. To elucidate the factors that interact with TMB, we further stratified patients based on tumor burden-related clinical parameters, including extranodal involvement, LDH, IPI, and stage, and the results demonstrate that in cases with higher tumor burden, more extranodal involvement sites, elevated LDH, advanced stage, higher IPI score, and higher mutation burden might be worse for the prognosis of these subsets of cases, indicating that mutation burden and tumor burden act as doubly impaired factors for survival. HBV-infection contributes to mutagenesis and is associated with poor prognosis for DLBCL patients [31, 32]; thus, we stratified cases based on HBV-infection. In HBV-infected patients, higher panel-TMB had no impact on survival, suggesting that HBV might act as an adverse factor but had no effect on the mutation burden induced by the virus. Studies have shown that TMB is significantly positively correlated with age in solid tumors, especially in patients with over 60 years of age [33]. However, little is known the prognostic importance between TMB in patients with age in DLBCL patients, in this study, we showed that higher panel-TMB was associated with a poor OS for younger than 60, while the level of panel-TMB was not significantly correlated with OS for greater than 60 years of age. The difference may be related to the characteristic of the malignancies, particularly the disorder in immune system.

To further shorten the gene panel, we used univariate COX regression to analyze the contribution of the NsMs of the 69 genes to OS in panel-TMB. We found that a panel of 13 genes was associated with poor OS, and another panel of 26 genes was correlated with the favorable OS, indicating that mutations in these 39 genes could be used to calculate panel-TMB and conduct prognostic stratification of DLBCL patients. MYD88, CREBBP, MYC, and FAT1 particularly attracted our attention because, in addition to their greater contribution to OS, their mutation frequency was greater than 10%. Studies have shown that MYD88, CREBBP, and MYC mutations play a vital role in regulating apoptosis/cell proliferation and transcription and predict adverse clinical outcomes in DLBCL patients [34–36], which is consistent with our results. Besides, a previous study has suggested that FAT1 is a tumor suppressor or has carcinogenic effects and participates in the regulation of cell metastasis [37], but this study demonstrates that FAT1 mutation contributes to the favorable OS for DLBCL patients. These results indicate that the NsMs in MYD88, CREBBP, MYC, and FAT1 significantly contribute to panel-TMB and prognostic stratification of DLBCL patients.

The limitation in this study is that the sample size may make panel-TMB statistically biased in predicting the OS of DLBCL patients. Moreover, we have not conducted clinical trials to evaluate the immune response of DLBCL patients to ICB using panel-TMB. To better describe the clinical value of panel-TMB in DLBCL patients, further investigation is needed.

## Conclusions

Herein, we reveal for the first time that the panel-TMB measured by GP69 could replace wTMB, and higher panel-TMB is associated with a poor OS for younger than 60, elevated LDH, greater than one extranodal involvement, stage III/IV, IPI score 3–5, and HBsAg, anti-HBc, or HBV-DNA negativity. Furthermore, nomogram constructed by panel-TMB, stage, and IPI could individually and visually predict the OS rates of DLBCL. Panel-TMB might be a potential predictor for the prognostic stratification of Chinese DLBCL patients.

## Supplementary Information

**Additional file 1: Table S1.** The description of 69 DLBCL-associated genes.

**Additional file 2: Fig. S1.** The biological processes (BPs) of the 69 genes in the panel were obtained from the Database for Annotation, Visualization, and Integrated Discovery (DAVID, https://david.ncifcrf.gov/). The area of each BP in the mosaic represents the number of genes, where the larger the area, the greater the number of genes.

**Additional file 3: Fig. S2.** The optimal cut-off values for panel-TMB in the GDPH **(A)** and TCGA **(B) **datasets.

**Additional file 4: Fig. S3.** Groups of genes with a coefficient > 0 or coefficient < 0 in univariate COX regression analysis in the GDPH and TCGA datasets. The histogram shows the number of overlapping genes (left panel). The circos plot shows the groups of overlapping genes (right panel). Genes with red color have a mutation frequency greater than 10%.

**Additional file 5: Fig. S4.** Kaplan-Meier survival analysis of panel-TMB calculated by 39 genes identified as prognosis-related genes in both the GDPH **(A)** and TCGA** (B)** datasets. The optimal cut-off values were obtained (left panel). Kaplan-Meier curves were plotted (right panel).

**Additional file 6: Fig. S5.** Subgroup analysis of panel-TMB in DLBCL patients of different genders, subtype, ECOG PS, and DHL/DEL. ECOG PS, Eastern Cooperative Oncology Group performance status; DHL/DEL, double-hit, and double-expressor lymphomas.

**Additional file 7: Fig. S6.** Kaplan–Meier survival analysis by the Ann Arbor stage classification, IPI score, gender, age, LDH, extranodal involvement, ECOG PS, subtype, DHL/DEL, HBsAg, anti-HBc, and HBV-DNA, respectively.

**Additional file 8: Table S2.** Points and OS rates in nomogram model.

## Data Availability

The TCGA-DLBCL data in this study were acquired using the UCSC XENA platform (https://xenabrowser.net/datapages/). The data that support the findings of this study are available from the corresponding author upon reasonable request.

## Abbreviations

Anti-HBc: Antibody to hepatitis B core antigen
CGP: Cancer gene panels
DLBCL: Diffuse large B-cell lymphoma
GP69: A panel of 69 genes
DHL/DEL: Double-hit: and double-expressor lymphomas
ECOG PS: Eastern Cooperative Oncology Group performance status
GCB: Germinal center B cells
HBsAg: Hepatitis B surface antigen
HBV-DNA: Hepatitis B virus DNA
IPI: International prognostic index
ICB: Immune checkpoint blockade
LDH: Lactate dehydrogenase
NGS: Next-generation sequencing
OS: Overall survival
panel-TMB: The tumor mutation burden calculated by a panel of 69 DLBCL-associated genes
TCGA: The Cancer Genome Atlas
TMB: Tumor mutation burden
WES: Whole-exome sequencing
wTMB: The tumor mutation burden calculated by whole-exome sequencing
