# Supplementary material for: Tumor mutation burden estimated by a 69-gene-panel is associated with overall survival in patients with diffuse large B-cell lymphoma
Source: Exp Hematol Oncol. 2021 Mar 15;10:20. doi: 10.1186/s40164-021-00215-4 (PMC7962318; doi:10.1186/s40164-021-00215-4)
Supplement: Supplementary file 1 — Additional file 1: Table S1. The description of 69 DLBCL-associated genes. [file 40164_2021_215_MOESM1_ESM.docx]

**Table S1.** The description of 69 DLBCL-associated genes.

| Gene symbol | Description |
| --- | --- |
| BCL10 | BCL10 immune signaling adaptor |
| SGK1 | serum/glucocorticoid regulated kinase 1 |
| NOTCH2 | notch receptor 2 |
| NFKBIE | NFKB inhibitor epsilon |
| FOXO1 | forkhead box O1 |
| PTEN | phosphatase and tensin homolog |
| TP53 | tumor protein p53 |
| EP300 | E1A binding protein p300 |
| MYD88 | MYD88 innate immune signal transduction adaptor |
| CXCR4 | C-X-C motif chemokine receptor 4 |
| TNFAIP3 | TNF alpha induced protein 3 |
| ERBB4 | erb-b2 receptor tyrosine kinase 4 |
| ROS1 | ROS proto-oncogene 1, receptor tyrosine kinase |
| FAS | Fas cell surface death receptor |
| BRAF | B-Raf proto-oncogene, serine/threonine kinase |
| KDR | kinase insert domain receptor |
| CREBBP | CREB binding protein |
| CARD11 | caspase recruitment domain family member 11 |
| NF1 | neurofibromin 1 |
| KRAS | KRAS proto-oncogene, GTPase |
| CD70 | CD70 molecule |
| MTOR | mechanistic target of rapamycin kinase |
| STAT6 | signal transducer and activator of transcription 6 |
| INPP5D | inositol polyphosphate-5-phosphatase D |
| DDX3X | DEAD-box helicase 3 X-linked |
| MEF2B | myocyte enhancer factor 2B |
| DTX1 | deltex E3 ubiquitin ligase 1 |
| TBL1XR1 | TBL1X receptor 1 |
| WHSC1 | Wolf-Hirschhorn syndrome candidate 1 |
| NFKBIE | NFKB inhibitor epsilon |
| MED12 | mediator complex subunit 12 |
| PAX5 | paired box 5 |
| IRF4 | interferon regulatory factor 4 |
| EBF1 | EBF transcription factor 1 |
| XPO1 | exportin 1 |
| SPEN | spen family transcriptional repressor |
| MYC | MYC proto-oncogene, bHLH transcription factor |
| ID3 | inhibitor of DNA binding 3, HLH protein |
| ATM | ATM serine/threonine kinase |
| CCND3 | cyclin D3 |
| CCND2 | cyclin D2 |
| PIM1 | Pim-1 proto-oncogene, serine/threonine kinase |
| TET2 | tet methylcytosine dioxygenase 2 |
| ATR | ATR serine/threonine kinase |
| ARID2 | AT-rich interaction domain 2 |
| KMT2D | lysine methyltransferase 2D |
| SMARCA4 | SWI/SNF related, matrix associated, actin dependent regulator of chromatin, subfamily a, member 4 |
| ARID1A | AT-rich interaction domain 1A |
| KDM6A | lysine demethylase 6A |
| KMT2A | lysine methyltransferase 2A |
| KMT2C | lysine methyltransferase 2C |
| CIITA | class II major histocompatibility complex transactivator |
| CD274 | CD274 molecule |
| TNFRSF14 | TNF receptor superfamily member 14 |
| PRDM1 | PR/SET domain 1 |
| CD58 | CD58 molecule |
| B2M | beta-2-microglobulin |
| PTPN6 | protein tyrosine phosphatase non-receptor type 6 |
| KLHL6 | kelch like family member 6 |
| PLCG2 | phospholipase C gamma 2 |
| CD79B | CD79b molecule |
| BCL2 | BCL2 apoptosis regulator |
| BTK | Bruton tyrosine kinase |
| LYN | LYN proto-oncogene, Src family tyrosine kinase |
| JAK3 | Janus kinase 3 |
| STAT3 | signal transducer and activator of transcription 3 |
| SOCS1 | suppressor of cytokine signaling 1 |
| GNA13 | G protein subunit alpha 13 |
| FAT1 | FAT atypical cadherin 1 |
