# Supplementary figures and images for: Tumor mutation burden estimated by a 69-gene-panel is associated with overall survival in patients with diffuse large B-cell lymphoma
Source: Exp Hematol Oncol. 2021 Mar 15;10:20. doi: 10.1186/s40164-021-00215-4 (PMC7962318; doi:10.1186/s40164-021-00215-4)

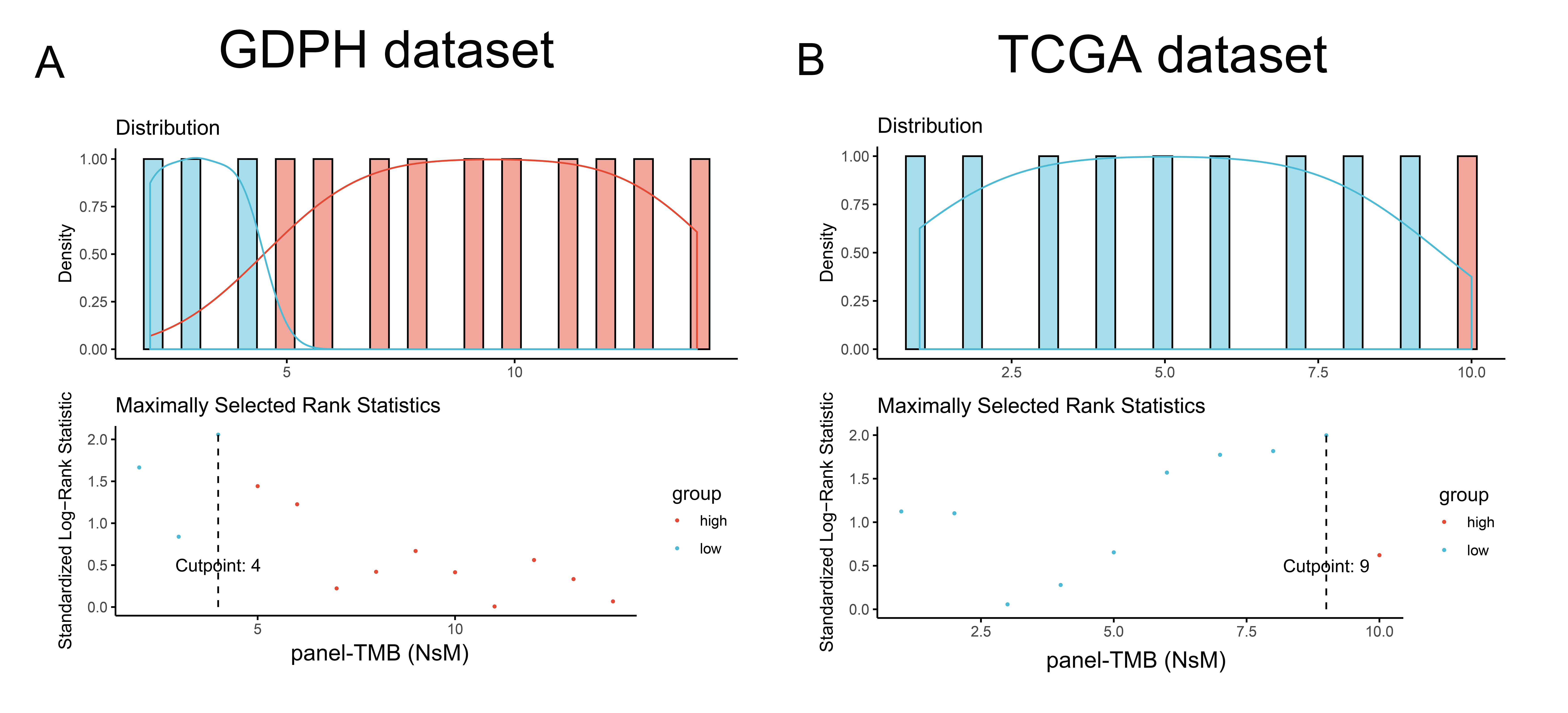

Supplement: Supplementary file 3 — Additional file 3: Fig. S2. The optimal cut-off values for panel-TMB in the GDPH (A) and TCGA (B) datasets. [file 40164_2021_215_MOESM3_ESM.tif]

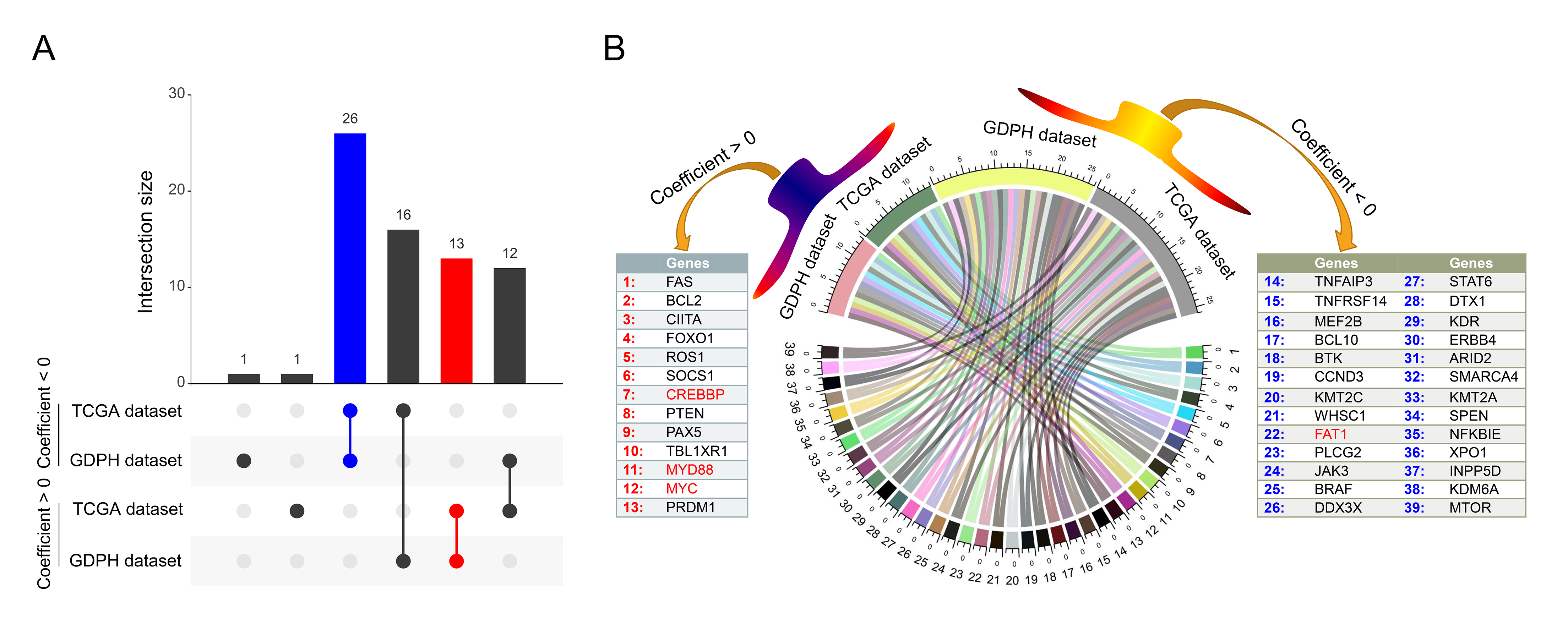

Supplement: Supplementary file 4 — Additional file 4: Fig. S3. Groups of genes with a coefficient > 0 or coefficient < 0 in univariate COX regression analysis in the GDPH and TCGA datasets. The histogram shows the number of overlapping genes (left panel). The circos plot shows the groups of overlapping genes (right panel). Genes with red color have a mutation frequency greater than 10%. [file 40164_2021_215_MOESM4_ESM.tif]

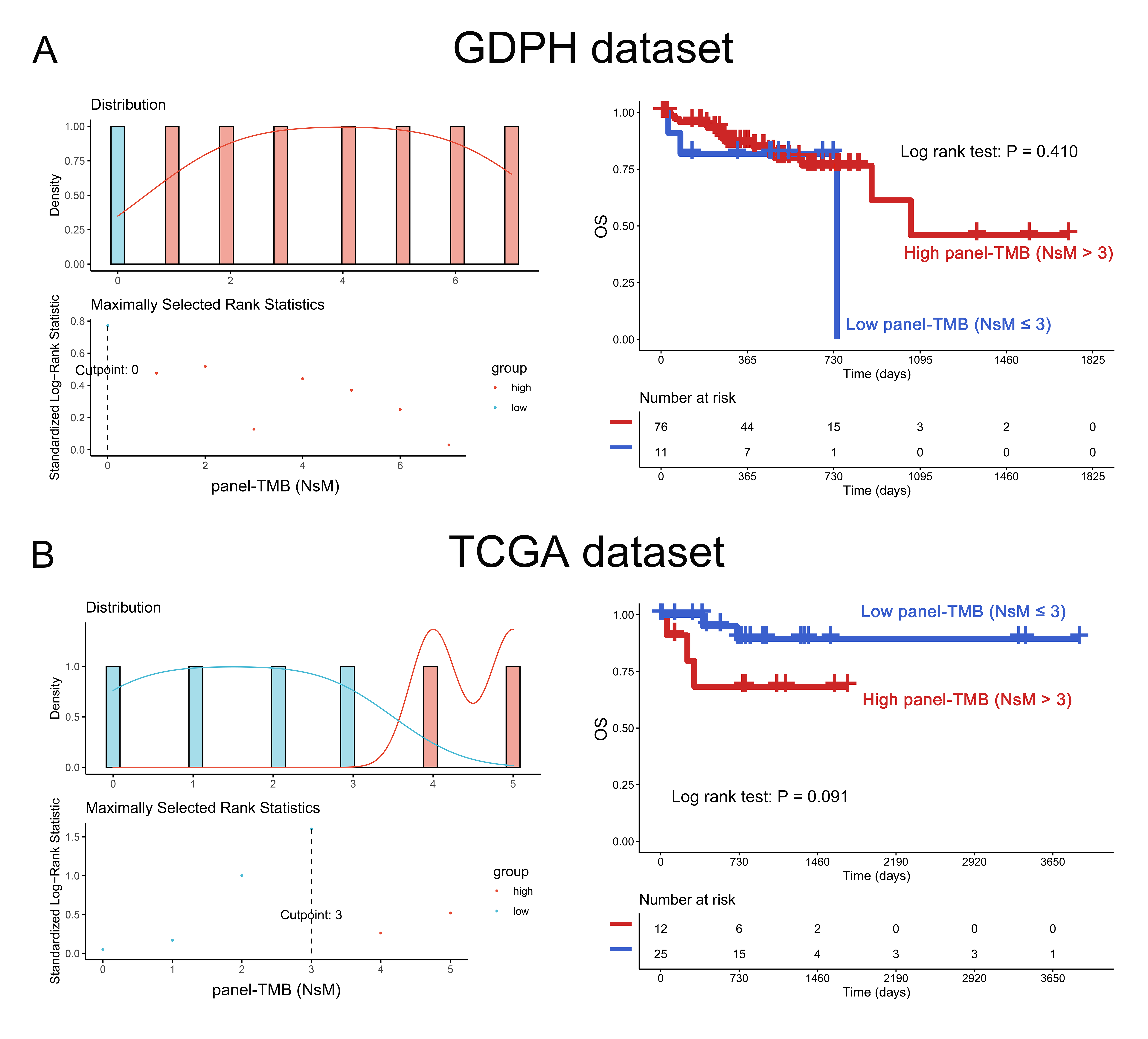

Supplement: Supplementary file 5 — Additional file 5: Fig. S4. Kaplan-Meier survival analysis of panel-TMB calculated by 39 genes identified as prognosis-related genes in both the GDPH (A) and TCGA (B) datasets. The optimal cut-off values were obtained (left panel). Kaplan-Meier curves were plotted (right panel). [file 40164_2021_215_MOESM5_ESM.tif]
