# Supplementary material for: Tumor mutation burden estimated by a 69-gene-panel is associated with overall survival in patients with diffuse large B-cell lymphoma
Source: Exp Hematol Oncol. 2021 Mar 15;10:20. doi: 10.1186/s40164-021-00215-4 (PMC7962318; doi:10.1186/s40164-021-00215-4)
Supplement: Supplementary file 8 — Additional file 8: Table S2. Points and OS rates in nomogram model. [file 40164_2021_215_MOESM8_ESM.doc]

**Table S2. Points and OS rates in nomogram model.**

| **Variables** | **Points** |  | **Survival time** | **OS rate** | **Total points** |
| --- | --- | --- | --- | --- | --- |
| Panel-TMB |  |  | 1 year | 0.95 | 87 |
| low | 0 |  |  | 0.90 | 143 |
| high | 100 |  |  | 0.85 | 178 |
| Ann Arbor stage |  |  |  | 0.80 | 203 |
| I/II | 0 |  |  | 0.70 | 240 |
| III/IV | 74 |  | 2 year | 0.95 | 42 |
| IPI |  |  |  | 0.90 | 99 |
| 0 - 2 | 0 |  |  | 0.85 | 133 |
| 3 - 5 | 67 |  |  | 0.80 | 158 |
|  |  |  |  | 0.70 | 195 |
|  |  |  |  | 0.60 | 223 |
|  |  |  |  | 0.50 | 248 |
|  |  |  | 3 year | 0.90 | 20 |
|  |  |  |  | 0.85 | 54 |
|  |  |  |  | 0.80 | 79 |
|  |  |  |  | 0.70 | 116 |
|  |  |  |  | 0.60 | 144 |
|  |  |  |  | 0.50 | 168 |
|  |  |  |  | 0.40 | 190 |
|  |  |  |  | 0.30 | 212 |
|  |  |  |  | 0.20 | 235 |

IPI, international prognostic index.
